# Public health round-up

**DOI:** 10.2471/BLT.23.011023

**Published:** 2023-10-01

**Devastation in Morocco**

Men stand on the rubble of destroyed homes in the village of Azro, near Tahanaout in the Atlas Mountains of Morocco. The country was hit by a powerful earthquake on 8 September. As of 14 September, 2497 people were reported to have been killed and 2476 injured.

**Figure:**
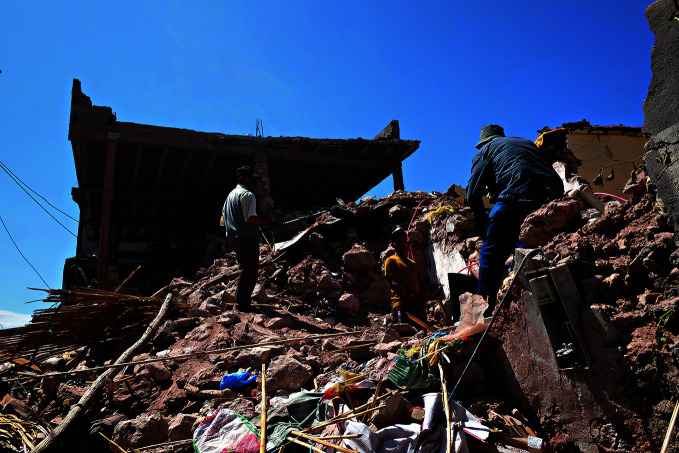
UNICEF/Brahim Benkirane

## Devastation in Libya

A storm system hit parts of the central and eastern Mediterranean, leading to devastating floods and massive loss of life in Libya.

Libya’s National Meteorological Centre reported that the storm, named Storm Daniel, reached a peak in northeastern Libya on 10 September, with winds up to 70–80 km/h and torrential rains that caused flooding in several cities. Worst hit was the coastal city of Derna, where waters from two burst dams caused widespread devastation.

The area affected is home to over 1.5 million people. As of 14 September, over 11 000 people were reported to have been killed while thousands more were reported missing. Over 35 000 people were reported to have been displaced, many of whom were left homeless.

The Permanent Mission of the State of Libya to the United Nations office in Geneva requested international assistance on 12 September. As of 14 September, the World Health Organization (WHO) had released 2 million United States dollars from its Contingency Fund for Emergencies, and was working with the Ministry of Health and partners to rush emergency relief to the affected areas.

WHO also activated a network of emergency medical teams and deployed contingency supplies that were already in the country. Some 28 metric tonnes of trauma, surgical and emergency supplies were sent from WHO’s logistics hub in Dubai.

https://bit.ly/46d0et7

https://bit.ly/3t1ObAr

https://bit.ly/3PlFAAp

## Morocco earthquake

A powerful earthquake (magnitude 6.8) struck Morocco on 8 September, the epicentre located 71 km south-west of Marrakesh in the High Atlas Mountains,

As of 13 September, Moroccan authorities had confirmed the loss of 2497 lives and the injury of 2476 people, with the number of casualties expected to increase. Most fatalities were reported in Al Haouz municipality, while the rest were reported in Ouarzazate, Azilal, Chichaoua and Taroudant provinces.

As of 14 September, the response was being led by government authorities supported by teams from the United Kingdom of Great Britain and Northern Ireland, Spain and Qatar.

The United Nations (UN) offered support with the situation assessment, coordination and response efforts, and WHO and its UN partners stood ready to scale up their response to provide supplies and technical assistance as needed.

https://bit.ly/46d0et7

https://bit.ly/45PtTJe

## Health at UNGA 78

WHO called on leaders scheduled to meet at the 78th session of the United Nations General Assembly (UNGA 78) in New York from 18 to 26 September, to put Health for All on the political agenda and apply lessons learned from the COVID-19 pandemic.

This year’s UNGA included an unprecedented number of health-focused meetings aimed at strengthening pandemic prevention, preparedness and response, delivering universal health coverage and ending the tuberculosis epidemic.

The UN also convened the Sustainable Development Goals Summit (SDG Summit), which was held from 18–19 September, and presented members with an opportunity to assess progress towards the 17 sustainable development goals.

In a 12 September media release, WHO stated that UNGA 78 was an opportunity to demonstrate that health is an investment, not a cost, and is fundamental to thriving, resilient families, societies and economies. “If COVID-19 taught us nothing else, it’s that when health is at risk, everything is at risk,” said WHO Director-General, Tedros Adhanom Ghebreyesus.

https://bit.ly/3Lpe57B

## Sharing COVID-19 technology

The COVID-19 Technology Access Pool (C-TAP), a multi-stakeholder partnership working to facilitate the sharing of intellectual property, knowledge and innovations, announced three new licensing agreements acquired through the Medicines Patent Pool, a United Nations-backed public health organization working to increase access to medical technology in low- and middle-income countries.

Announced on 29 August, the agreements cover a COVID-19 vaccine, a second license for a COVID-19 vaccine prototype, and technology for a COVID-19 assay.

“COVID-19 is here to stay, and the world will continue to need tools to prevent it, test for it and treat it,” said WHO Director-General, Tedros Adhanom Ghebreyesus in a 29 August media release. 

https://bit.ly/44MmkBC

## New digital health initiative

WHO and the G20 India Presidency announced a new Global Initiative on Digital Health (GIDH).

Announced on 19 August at the Health Ministers’ Meeting of the G20 Summit hosted by the Government of India, the initiative will operate as a WHO-managed network and platform to support the implementation of the *Global strategy on digital health 2020–2025*.

WHO serves as the Secretariat for the strategy, working to support global implementation, the establishment of global standards best practices, and mobilisation of resources needed to fast track digital health system transformation.

https://bit.ly/3EFpRHu

## Collaborating with civil society

WHO launched the WHO Civil Society Commission to facilitate more structured and systematic dialogue between WHO and civil society on health priorities and related issues. 

Launched on 24 August, the Commission was created in response to civil society requests to explore better and more meaningful ways to engage with WHO, above and beyond those which already exist. As of 24 August, over 350 organizations had applied to be part of the Commission, 120 of which had been accepted. The list of participants can be found on the WHO website and will be regularly updated.

https://bit.ly/3Ljt6Yv

## Human papillomavirus in men

Just under a third of all men over the age of 15 are infected with at least one genital human papillomavirus (HPV) type, and 1 in 5 are infected with one or more of what are known as high risk, or oncogenic, HPV types.

This is according to the results of a systematic review and meta-analysis assessing the prevalence of genital HPV infection in the general male population, based on studies published between 1995 and 2022.

Published in *The Lancet Global Health* on 1 September, the estimates show that men frequently harbour genital HPV infections, and underline the importance of incorporating men in efforts to control HPV infection and reduce the incidence of HPV-related disease in both men and women.

https://bit.ly/3Ln58f4

## Patient safety charter

A broad range of stakeholders agreed on a first-ever patient safety rights charter, which outlines the core rights of all patients and seeks to assist governments and other stakeholders to ensure that the voices of patients are heard and their right to safe health care is protected.

The agreement was reached at a WHO-hosted conference which was held on 12 and 13 September and was attended by more than 2300 people from all six WHO regions, including patient advocates and representatives of patients’ organizations.

https://bit.ly/3EEws4I

**Cover photo**

A cholera vaccinator shows a boy how to wash his hands to protect himself against cholera infection at a church in Mashuuru, Kajiado, Kenya.

**Figure:**
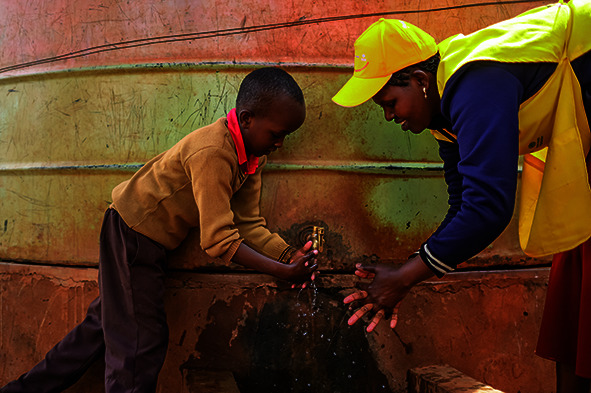
(WHO/Billy Miaron)

## Tools for knowledge translation

WHO launched an online repository of evidence-informed decision-making tools. Launched on 24 August, the repository is the first of its kind to promote access to the knowledge translation tools used by WHO and partner organizations involved in planning, managing, monitoring and evaluating the process of evidence use and implementation.

The easy-to-navigate online platform enables users to search and access methods and tools corresponding to each step of the policy/action cycle.

https://bit.ly/3EAv4jL

## New suicide prevention resources

WHO released two new resources designed to support suicide prevention efforts. The first, *Preventing suicide: a resource for media professionals*, produced in collaboration with the International Association for Suicide Prevention, presents current evidence on the impact of media reporting of suicide and provides practical guidance for media professionals on how to report on suicide responsibly.

The second is a policy brief focused on the public health implications of suicide decriminalization. Drawing on the experiences of countries that have recently decriminalized suicide and suicide attempts, including Guyana, Pakistan and Singapore, the brief sets out recommendations for policy-makers, legislators and other decision-makers considering reform in this area.

https://bit.ly/3PFhimi

**Looking ahead**

5–6 October 2023. First meeting of the Technical Advisory Group on Genomics. WHO HQ, Geneva. https://bit.ly/3LpPXBT

5–6 October 2023. Global Research and Innovation Forum: Building the world’s resilience against future outbreaks and pandemics. WHO HQ. https://bit.ly/3PEI2mU

10–11 October 2023. Regional Summit for Policy Innovation on Healthy Ageing. Lisbon, Portugal. https://bit.ly/3RmHzqt

